# Dual-Isotope (δ^2^H, δ^18^O) and Bioelement (δ^13^C, δ^15^N) Fingerprints Reveal Atmospheric and Edaphic Drought Controls in Sauvignon Blanc (Orlești, Romania)

**DOI:** 10.3390/plants14243816

**Published:** 2025-12-15

**Authors:** Marius Gheorghe Miricioiu, Oana Romina Botoran, Diana Costinel, Ionuț Făurescu, Roxana Elena Ionete

**Affiliations:** National Research and Development Institute for Cryogenic and Isotopic Technologies—ICSI Ramnicu Vâlcea, 4th Uzinei Street, 240050 Ramnicu Vâlcea, Romania; marius.miricioiu@icsi.ro (M.G.M.); diana.costinel@icsi.ro (D.C.); ionut.faurescu@icsi.ro (I.F.); roxana.ionete@icsi.ro (R.E.I.)

**Keywords:** stable isotopes, δ^18^O, δ^2^H, δ^13^C, δ^15^N, drought, viticulture, soil moisture, isotopic drought index

## Abstract

Grapevine water relations are increasingly influenced by drought under climate change, with significant implications for yield, fruit composition and wine quality. Stable isotopes of hydrogen, oxygen, carbon and nitrogen (δ^2^H, δ^18^O, δ^13^C and δ^15^N) provide sensitive tracers of plant water sources and physiological responses to stress. Here, we combined dual water isotopes (δ^2^H, δ^18^O), carbon and nitrogen isotopes (δ^13^C, δ^15^N), and high-resolution micrometeorological/soil observations to diagnose drought dynamics in *Vitis vinifera* cv. Sauvignon blanc (Orlești, Romania; 2023–2024). Dual-isotope relationships delineated progressive evaporative enrichment along the soil–plant–atmosphere continuum, with slopes LMWL ≈ 6.41 > stem ≈ 5.0 > leaf ≈ 2.2, consistent with kinetic fractionation during transpiration (leaf) superimposed on source-water signals (stem). Weekly leaf δ^18^O covaried strongly with relative humidity (RH; r = −0.69) and evapotranspiration (ET; r = +0.56), confirming atmospheric control of short-term enrichment, while stem isotopes showed buffered responses to soil water. We integrated Δ^18^O (leaf–stem), RH, ET, and soil matric potential at 60 cm (Soil_60_) into an Isotopic Drought Index (IDI), which captured the onset, intensity, and persistence of the July–August 2024 drought (IDI_0–100_ > 90; RH < 60%, ET > 40 mm wk^−1^, Soil_60_ > 100 cb). Carbon and nitrogen isotopes provided complementary, integrative diagnostics: δ^13^C increased (less negative) with drought (r = −0.52 with RH; +0.49 with IDI), reflecting higher intrinsic water-use efficiency, whereas δ^15^N rose with soil dryness and IDI (leaf: r ≈ +0.48 with Soil_60_; +0.42 with IDI), indicating constraints on N acquisition and enhanced internal remobilization. Together, multi-isotope and environmental data yield a mechanistic, field-validated framework linking atmospheric demand and edaphic limitation to vine physiological and biogeochemical responses and demonstrate the operational value of an isotope-informed drought index for precision viticulture.

## 1. Introduction

Most crops are affected by biotic and abiotic stresses during the growing and harvesting seasons, and these stresses may be reflected in the quality of the final product—as is the case for wine. Although the grapevine (*Vitis vinifera* L.) is adapted to a wide range of pedoclimatic conditions, its physiological performance can be compromised under abiotic stress, especially drought. Drought reduces photosynthetic activity, stomatal conductance and canopy transpiration, which in turn may lead to lower yields and altered berry composition [1]. During the vegetative period, plant water use varies markedly, depending on meteorological conditions, soil water availability and plant-water relations [2]. In viticulture, water deficit not only affects vine growth and yield but also influences the accumulation of primary and secondary metabolites in grapes, thereby impacting wine organoleptic characteristics [3].

Climate change is shifting the hydrological balance of temperate viticultural regions, leading to more frequent and intense drought episodes that threaten vineyard productivity and quality. In this context, understanding the exchanges among plant, soil and atmosphere in the vineyard system is critical for sustainable management of water resources [4]. The monitoring of vine-water relations under changing climatic conditions is thus imperative.

Stable isotope analysis has become a cornerstone of ecohydrology and plant physiology, providing direct tracers of water sources and evaporative/transpirative processes in the plant–soil–atmosphere continuum [5]. The stable isotope composition of hydrogen (^2^H) and oxygen (^18^O) in plant water reflects isotopic fractionation during evaporation and transpiration and thus records prevailing environmental conditions during water uptake and movement [6]. In particular, the relationship between δ^2^H and δ^18^O (often assessed in the dual-isotope space) allows assessment of evaporative enrichment and deviations from the Global Meteoric Water Line (GMWL; δ^2^H = 8 × δ^18^O + 10) under the influence of relative humidity, temperature and stomatal control [7].

In dual δ^2^H–δ^18^O space, precipitation typically plots along the Global Meteoric Water Line, a relationship first described by Craig (1961) and resulting from equilibrium fractionation during condensation and re-evaporation processes in the atmosphere [8]. At the regional scale, a Local Meteoric Water Line (LMWL) emerges from the isotopic composition of precipitation and often displays slopes lower than 8 due to sub-cloud evaporation and local climatic influences [9]. Within plants, stem and leaf water typically deviate from meteoric lines and form “leaf water lines” with lower slopes, reflecting progressive evaporative enrichment driven by both equilibrium and kinetic fractionation at the evaporative sites of the leaf, as originally formulated by Dongmann et al. (1974) [10] and later refined by Gat (1996) [5] and Farquhar et al. (2020) [11]. These dual-isotope relationships provide a physical basis for distinguishing between source-water signals (precipitation, xylem) and evaporative modification within plant tissues. Many studies have adapted the classical Craig–Gordon model to characterize leaf-water isotopic composition, although it was originally developed for ocean-surface evaporation processes [8]. In grapevines, isotopic signatures in stem and leaf water allow inference of water uptake depth, root activity, transpiration rates and stomatal responses under water-limited conditions [6,12,13].

In parallel, carbon and nitrogen stable isotopes provide complementary insights into plant physiological state. The enzyme Rubisco discriminates against ^13^C during CO_2_ fixation, so organic material typically becomes depleted in ^13^C relative to the atmosphere [14]. Under drought conditions, stomatal closure restricts CO_2_ diffusion into the leaf, reducing discrimination and thereby leading to more positive δ^13^C values, which correlate with increased intrinsic water-use efficiency (iWUE) [15]. Likewise, δ^15^N reflects soil–plant nitrogen cycling and may be altered under drought due to changes in microbial nitrification, root uptake and internal remobilisation, thus serving as an indicator of nitrogen status under water stress [16,17]. While each of these isotope systems (δ^2^H, δ^18^O, δ^13^C, δ^15^N) has been applied in viticultural science, only a limited number of studies have integrated them together with microclimate and soil moisture data to disentangle atmospheric vs. edaphic drought influences on the vine [18,19]. In practice, most isotope work in viticulture has relied on δ^13^C in must, berries or leaves as an integrated indicator of vine water status and intrinsic water-use efficiency, sometimes complemented by δ^15^N to gain information on nitrogen supply and fertilisation regime. Dual water isotopes (δ^2^H, δ^18^O) have been used more often to characterise wine origin and broad climatic conditions than to resolve vine water uptake and evaporative drivers at the plot scale. By contrast, ecohydrological studies in forests and other ecosystems increasingly combine δ^2^H–δ^18^O in soil and xylem water with micrometeorological data to distinguish atmospheric demand from soil water limitation and to track changes in rooting depth.

Vineyard-scale studies that adopt this type of ecohydrological framework are still uncommon. In particular, there is little work that brings together (i) dual water isotopes in precipitation, stem and leaf water, (ii) continuous microclimate records, (iii) depth-resolved soil water status, and (iv) δ^13^C and δ^15^N as markers of the longer-term physiological and nutritional consequences of drought. To our knowledge, no previous study has combined all four isotope systems (H, O, C, N) with high-resolution meteorological and soil matric potential measurements in vineyards in order to explicitly separate atmospheric and edaphic drought phases and to build a process-based isotopic drought index.

In this study, we address this gap with an integrated isotopic investigation of *V. vinifera* cv. Sauvignon Blanc in the Orlești vineyard (Vâlcea County, Romania). The following working hypotheses at the vineyard scale were tested: (i) dual water isotopes (δ^2^H, δ^18^O) in precipitation, stem and leaf water show a progressive enrichment from source water to foliage, and leaf δ^18^O/Δ^18^O are tightly coupled to short-term atmospheric drought (RH, ET); (ii) stem water isotopes and soil matric potential at depth (Soil_60_) reflect slower, edaphic control on water availability and can be integrated into a process-based Isotopic Drought Index (IDI); and (iii) δ^13^C and δ^15^N in leaves and stems respond to the cumulative intensity of seasonal drought (IDI, Soil_60_) and differ between years, acting as integrative indicators of carbon and nitrogen stress. In doing so, we aim to provide a vineyard-scale, isotope-based framework that can be used to diagnose both rapid atmospheric stress and cumulative soil water limitation under increasingly variable climate conditions.

## 2. Materials and Methods

### 2.1. Study Site and Experimental Design

**Figure 1:**
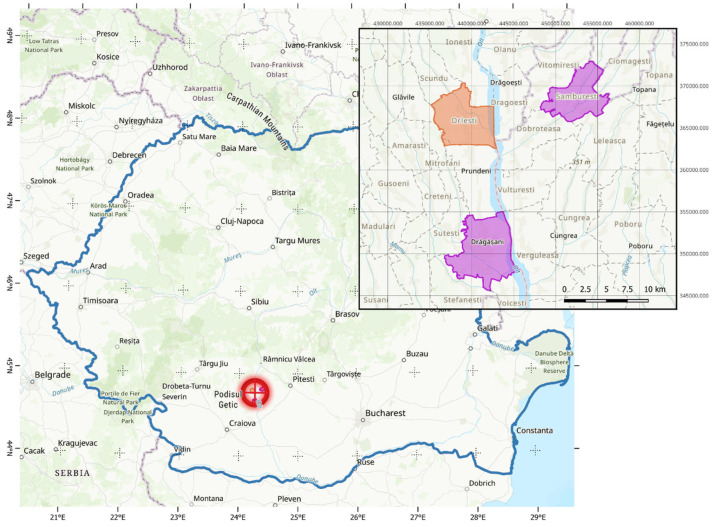
Location of experimental site (Orleşti) near two traditional wine making regions (Drăgășani and Sâmburești).

The study was conducted in the Orlești vineyard, Vâlcea County, Romania (44°39′ N, 24°10′ E), located within the traditional viticultural region of Drăgășani–Sâmburești along the Olt River Valley (Romania)—see Figure 1. The area has a temperate-continental climate, with warm summers and moderate annual precipitation (approximately 620 mm). The soil is a clay–loam luvisol typical of Romanian vineyard soils. The vineyard is non-irrigated, and rainfall was the only water input during the 2023–2024 study period. The canopy was maintained at approximately 1.2 m^2^ per vine through trellis constraints and routine green pruning. The soil was ploughed in spring, and no mineral fertilizers, organic manures or other nitrogen-containing amendments were applied during the study years. These management conditions minimise external inputs that could affect δ^13^C and δ^15^N, allowing us to focus on the influence of water availability and climate on the isotopic signatures.

Weekly sampling campaigns were carried out during the 2023 and 2024 growing seasons, covering the main phenological stages of *Vitis vinifera* cv. Sauvignon blanc. Samples of leaves and stems were collected from representative vines between May and October. Precipitation samples were also collected monthly to establish the Local Meteoric Water Line (LMWL) for the study area.

### 2.2. Meteorological and Soil Monitoring

**Figure 2:**
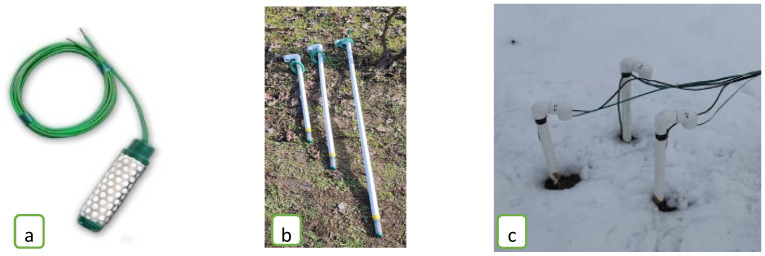
(**a**) Humidity sensor; (**b**,**c**), positioning of humidity sensors in the vineyard.

Meteorological parameters were continuously recorded using a DAVIS Vantage Pro2 (Davis Instruments, Hayward, CA, USA) automatic weather station installed in the vineyard. The system measured air temperature, relative humidity, wind speed, atmospheric pressure, precipitation, and evapotranspiration (ET) at 30 min intervals (Figure 2).

Soil temperature and moisture were monitored using three brand new Davis Instruments Soil Moisture Sensors (Model 6440, Davis Instruments, Hayward, CA, USA) coupled with three brand new Davis Instruments Stainless Steel Temperature Probes (Model 6470) positioned at depths of 30, 60 and 100 cm. Soil temperature sensors operate based on the Seebeck effect, in which two dissimilar conductors or semiconductors form junctions maintained at different temperatures. The temperature gradient between these junctions generates a voltage difference (the Seebeck voltage) proportional to the temperature difference, which can be measured to determine temperature precisely.

Davis Instruments Soil Moisture Sensors measure soil water tension (matric potential) in centibars (cb), providing an indirect indication of water availability to plants. The sensors are robust, easy to use and cost-effective, with a stainless-steel casing that protects them from corrosion during prolonged exposure to moist soil. They are unaffected by freezing temperatures and have a wide measurement range (0–200 cb), allowing accurate operation under both wet and dry conditions. Before installation, each sensor was verified against the manufacturer’s factory calibration and published protocols [20] by testing in media with known moisture conditions to confirm an appropriate response curve. The 6470 probe provided automatic temperature compensation to correct for the influence of soil temperature on sensor output. Throughout the growing season, sensors were periodically inspected for stability, and readings were cross-checked under consistent soil conditions to detect potential measurement drift. No significant drift was observed, and sensor performance remained within the expected range for field monitoring. Soil temperature and moisture were recorded automatically at 30 min intervals and aggregated as weekly averages to match isotope sampling dates.

In the following, we use the term atmospheric drought to describe periods of high evaporative demand, driven by low relative humidity (RH), high evapotranspiration (ET) and elevated air temperature, even when soil water availability is still relatively high. By edaphic drought we refer to limitations in water supply within the root zone, as indicated by high soil matric potential (Soil_60_), regardless of short-term fluctuations in RH or ET. In this framework, RH and ET are primarily used to characterise the atmospheric component of drought, whereas Soil_60_ captures the soil-based (edaphic) component that directly constrains vine water uptake.

### 2.3. Sample Collection and Isotopic Analysis

Precipitation, leaf, and stem samples were collected during the 2023–2024 growing seasons in the Orlești vineyard (Vâlcea County, Romania).

*Precipitation sampling.* Precipitation was collected using a rain gauge equipped with a thin mineral oil layer to prevent post-collection evaporation and isotopic fractionation (IAEA-GNIP protocol, 2014). Immediately after each rainfall event, samples were transferred into 30 mL glass vials, sealed with Parafilm, and stored at 4 °C until analysis. These samples were used to establish the local meteoric water line and to interpret isotopic enrichment in vine water.

*Plant sampling.* Leaves and stems of *Vitis vinifera* L. cv. Sauvignon blanc were collected weekly from representative vines across the vineyard during the 2023–2024 growing seasons, always at the same time of day to minimise diurnal variability in isotopic composition. This sampling frequency was selected to resolve seasonal changes in vine water status and isotope composition across the main phenological stages, rather than short-lived post-event fluctuations after individual rainfall or irrigation events. Bulk leaf and stem isotopic signals integrate environmental and physiological conditions over several days, so a weekly resolution is adequate to track the development of drought and to compare years with contrasting water availability. Meteorological and soil variables were recorded continuously (30 min interval) and then averaged to the sampling dates, allowing us to link isotopic measurements to the corresponding atmospheric and soil conditions while focusing on the seasonal evolution of drought. Immediately after collection, leaves and stems were placed into airtight bags, kept cool and in the dark, transported to the laboratory and lyophilised the same day to minimise post-harvest metabolic activity and isotopic exchange. Water was extracted from plant tissues by lyophilisation, and the extracted water was stored in sealed glass vials at 4 °C until analysis. These handling and extraction procedures were designed to avoid evaporation and contamination and to ensure that measured isotope ratios reflect in situ plant water.

*Hydrogen and oxygen isotope analysis (δ^*2*^H, δ^*18*^O).* Water extracted from plant tissues and precipitation samples were analyzed using a DELTA V Plus Continuous-Flow Isotope Ratio Mass Spectrometer (CF-IRMS) (Thermo Fisher Scientific, Bremen, Germany) coupled to a GasBench II isotope equilibration module. Data were processed with *Isodat 3.0* software. For δ^18^O analysis, 500 µL of sample was equilibrated for 20 h at 24 °C with a 0.36% CO_2_–He gas mixture; ^18^O/^16^O ratios were determined from m/z 46 and 44 ion currents. For δ^2^H, 200 µL of water was equilibrated with a 2% H_2_–He mixture in the presence of a platinum catalyst for 1 h at 25 ± 0.2 °C, and ^2^H/^1^H ratios were measured from m/z 3 and 2 ion currents.

Results were expressed in delta notation (δ, ‰) relative to VSMOW, using the relationship

$$\delta_{sample}\left(‰\right)=\left[\frac{R_{sample}}{R_{standard}}-1\right]\times 1000$$

Analyses were normalized with in-house water standards calibrated against IAEA primary references (VSMOW2, SLAP2, GISP): B2192 (δ^2^H = +11.26 ± 1.34‰, δ^18^O = −0.41 ± 0.11‰), IA-R064 (δ^2^H = −98.32 ± 1.13‰, δ^18^O = −12.34 ± 0.13‰), and OH32 (δ^2^H = +17.30 ± 0.70‰, δ^18^O = +1.22 ± 0.09‰). Analytical reproducibility was better than ± 0.2‰ for δ^18^O and ± 1‰ for δ^2^H.

*Carbon and nitrogen isotope analysis (δ^*13*^C, δ^*15*^N).* Oven-dried tissues were homogenized with a knife mill (GM 200, Retsch, Hahn, Germany). Aliquots of 2–3 mg were encapsulated in tin and analyzed using a Flash EA 1112 HT Elemental Analyzer coupled via continuous flow to a DELTA V Plus CF-IRMS (Thermo Scientific, Bremen, Germany). Combustion followed the Dumas method at 980 °C (C) and 960 °C (N), with helium 5.0 as carrier gas. Generated CO_2_ and N_2_ were diluted through a ConFlo III interface and introduced into the IRMS via a MAS200 autosampler.

Isotopic ratios were reported relative to VPDB (carbon) and AIR (nitrogen). Calibration and quality control used certified reference materials (Iso-Analytical Ltd., Crewe, UK): IA-R001 (wheat flour), IA-R004 (corn flour), IA-R041 (alanine) for δ^13^C, and IA-R001 (wheat flour) and IA-R045 (ammonium sulfate) for δ^15^N. Analytical precision was ± 0.40‰ for both isotopes. All measurements were traceable to international reference scales through calibration against IAEA primary standards (VSMOW2, SLAP2, GISP, VPDB, AIR).

### 2.4. Derived Isotope Metrics and Modelling

To disentangle the atmospheric and edaphic controls on vine water isotopic composition and to develop an integrative drought indicator, three derived isotope metrics were calculated: (i) the deuterium excess (*d-excess*) as a tracer of equilibrium versus kinetic fractionation, (ii) the isotopic enrichment above xylem water (Δ^18^O) as a proxy for evaporative fractionation at the leaf level, and (iii) the Isotopic Drought Index (IDI) as a composite measure combining atmospheric demand, soil water status, and plant isotopic response.

*Deuterium excess*—calculated in ‰ (VSMOW) following Dansgaard (1964) [21]: $d_{excess}=\delta^{2}H-8\cdot \delta^{18}O$. The *d-excess* quantifies the deviation from the Global Meteoric Water Line (GMWL) and reflects the balance between equilibrium and kinetic fractionation. Positive values near +10‰ indicate equilibrium conditions and high relative humidity, whereas strongly negative *d* values in leaf water denote pronounced kinetic fractionation during transpiration under low RH and high ET. In plant water, d-excess therefore carries information that is partly independent from bulk δ^2^H and δ^18^O. When equilibrium processes dominate (e.g., recent meteoric recharge, high relative humidity), d-excess in stem and soil water tends to remain close to meteoric values. When kinetic fractionation during transpiration becomes strong (low RH, high ET), leaf water shifts towards much more negative d-excess, even if the isotopic composition of the source water (xylem) changes only slightly. Following both stem and leaf d-excess thus helps to distinguish between changes driven mainly by atmospheric evaporative demand and those associated with shifts in the source-water pool accessed by roots.

*Isotopic enrichment above xylem* (Δ^18^O)—to isolate the evaporative enrichment occurring at the leaf level from the isotopic composition of stem (xylem) water, isotopic enrichment was defined as:

$${∆}^{18}O_{leaf-stem}=\delta^{18}O_{leaf}-\delta^{18}O_{stem}$$

Δ^18^O serves as a direct physiological proxy of atmospheric drought, controlled primarily by leaf boundary-layer conditions and the humidity of the surrounding air [8,16]. In our interpretation, Δ^18^O mainly reflects the rapid response of leaf water to changes in the atmospheric environment, because enrichment above the xylem source is controlled by RH, ET and boundary-layer conditions at the leaf surface. By contrast, the isotopic composition of stem water is expected to change more slowly and to integrate shifts in the mixture of soil water sources accessed by the roots as the profile dries, that is, the edaphic component of drought. This distinction underpins the separation between atmospheric and soil-driven processes in our subsequent analysis and in the construction of the IDI.

*Theoretical basis of dual-isotope relationships*—the interpretation of LMWL and leaf water lines in this study follows the classical Craig–Gordon evaporation model (Craig & Gordon, 1965) [8], which remains the foundation of isotopic studies in terrestrial vegetation. In its steady-state form, the bulk isotopic composition of leaf water (δ_L_) relative to its source water (xylem water; δ_S_) can be approximated as:

$$\delta_{L}\approx \delta_{S}+\varepsilon^{*}+\left(\varepsilon_{k}+\varepsilon^{*}\right)\left(1-h\right)$$

where $\varepsilon^{*}$ is the temperature-dependent equilibrium fractionation factor between liquid water and vapour [22], $\varepsilon_{k}$ is the kinetic fractionation associated with molecular diffusion through stomata and the leaf boundary layer [23], and h is the relative humidity normalized to leaf temperature. Under high humidity (h → 1), enrichment is minimal and δ_L_ approaches δ_S_. Conversely, under low humidity, the combined equilibrium and kinetic terms induce strong enrichment in both δ^18^O and δ^2^H, producing characteristic “evaporation lines” with slopes typically between 2 and 5 [24].

In our vineyard dataset, the hierarchy of slopes (LMWL ≈ 6.41 > stem ≈ 5.04 > leaf ≈ 2.24) matches expectations derived from Craig–Gordon theory, indicating progressive enrichment from meteoric inputs to xylem and finally to leaf water.

*Isotopic Drought Index* (IDI)—to integrate the concurrent atmospheric (RH, ET) and edaphic (soil matric potential) components of drought with the isotopic response (Δ^18^O), an Isotopic Drought Index (IDI) was constructed using standardized (z-score) variables:

$$z\left(x\right)=\frac{x-\bar{x}}{S_{x}}$$

where $\bar{x}$ and $s_{x}$ are the mean and standard deviation of each variable.

The composite raw index was defined as:

$${IDI}_{raw}=\frac{z\left({∆}^{18}O\right)+z\left(ET\right)+z\left({Soil}_{60}\right)-z(RH)}{4}$$

The negative sign for RH accounts for its inverse relationship with drought intensity (low RH = dry conditions). For comparison across years and visualization, IDI*_raw_* values were rescaled to a 0–100 dimensionless range using min–max normalization:

$${IDI}_{0-100}=\frac{{IDI}_{raw}-min({IDI}_{raw})}{\max\left({IDI}_{raw}\right)-min({IDI}_{raw})}$$

Equal weights were applied to each standardized variable because they represent distinct physical processes (isotopic enrichment, evaporative demand, soil water limitation, and atmospheric moisture). Weighted alternatives could be optimized using AIC-based regression or cross-validation, but the equal-weight configuration robustly reproduced the 2024 drought pattern (IDI_0–100_ > 90 in July–August).

Δ^18^O represents the rapid isotopic expression of atmospheric stress; ET and RH quantify the atmospheric drought component; and Soil_60_ integrates the edaphic drought component, that is, water availability in the active rooting zone—less variable than the shallow 30 cm layer yet more responsive than 100 cm. Soil_60_ was selected as the rooting-zone variable because grapevine fine roots are most abundant in the upper 60 cm and main roots typically extend between 18–80 cm, making this depth representative of the active water-uptake zone in this vineyard [5,6,7,8,9]. Together, these variables capture both short-term evaporative effects and slower soil-water feedback, producing a physiologically meaningful isotopic drought signal.

Scientific Basis and Weighting Structure of the IDI—The IDI was developed as a mechanistic composite indicator integrating four independent drivers of plant water status: (i) Δ^18^Oleaf–stem, which reflects leaf-level evaporative enrichment and thus the isotopic expression of atmospheric demand; (ii) relative humidity (RH) and evapotranspiration (ET), representing short-term atmospheric drought; and (iii) soil water tension (Soil_60_), capturing edaphic water limitation. These variables represent distinct physiological and environmental processes, each known to exert strong and non-redundant controls on isotope-based indicators of transpiration, stomatal regulation, and plant water availability [12,23,25]. Because IDI is intended as a process-based indicator rather than a predictive statistical model, traditional weight-optimization methods (e.g., AIC, BIC, machine-learning regressors) are not applicable in the absence of a defined target variable to optimize against (such as sap flow, predawn water potential, or yield). In this context, assigning differential weights would introduce arbitrary and non-transferable coefficients lacking physiological justification.

Therefore, and following established procedures for constructing composite ecohydrological indices [26,27], we adopted an equal-weighting approach, which ensures transparency, reproducibility, and independence from statistical model fitting.

To evaluate the robustness of this weighting structure, we performed a ±25% sensitivity analysis in which each variable’s contribution was perturbed relative to its equal weight. Across all scenarios, the seasonal trajectory of IDI, the relative drought ranking between years, and the detection of the major 2024 drought pulse remained unchanged (Supplementary Figure S1). This demonstrates that IDI is insensitive to moderate weighting adjustments and supports its suitability as an integrative, physiologically grounded indicator capable of distinguishing between atmospheric and edaphic drought phases in vineyards.

### 2.5. Statistical Analyses

All statistical analyses were carried out on weekly mean values for the 2023–2024 growing seasons. Environmental variables (T, RH, ET, soil matric potential), isotopic variables (δ^2^H, δ^18^O, Δ^18^O, d-excess, δ^13^C, δ^15^N), and the Isotopic Drought Index (IDI) were first inspected graphically using time-series plots, histograms and Q–Q plots to identify obvious outliers, non-linear patterns and clear deviations from approximate normality. Because most variables showed roughly symmetric distributions after aggregation to weekly scale, we used Pearson correlation coefficients (r) to quantify bivariate relationships. Reported *p*-values are based on two-tailed tests with a significance level of α = 0.05 and are interpreted in an exploratory way, given the moderate sample size and the number of comparisons.

To examine temporal structure, we inspected autocorrelation functions (ACF) for key variables and calculated simple lagged correlations (lags 1–2 weeks) between stem and leaf isotopes and Soil_60_. These analyses were used only to check whether isotopic responses showed a strong delayed behaviour relative to changes in soil water status, rather than to build formal time-series models. Independence between years was treated pragmatically by analysing the combined 2023–2024 weekly series, while also comparing patterns between the two seasons in the PCA and IDI time series.

Simple linear regressions (ordinary least squares) were used to derive the Local Meteoric Water Line (LMWL) and the leaf and stem “evaporation lines” (δ^2^H vs. δ^18^O), and to describe the main isotope–environment relationships discussed in the text (for example δ^18^O_leaf and Δ^18^O_leaf–stem vs. RH and ET, and δ^13^C and δ^15^N vs. Soil_60_ and IDI). For each regression we report slope and intercept, the coefficient of determination (R^2^).

Principal component analysis (PCA) was applied to standardised (z-score) variables to summarise the joint structure of isotopic, climatic and soil data. All variables were centred and scaled to unit variance before analysis so that loadings reflect correlation structure rather than differences in measurement units. Components with eigenvalues > 1 were retained for interpretation, and the first two axes (F1–F2) were used to describe the dominant drought gradient and the separation between atmospheric and edaphic influences.

The Isotopic Drought Index (IDI) was computed as described in Section 2.4 from z-standardised Δ^18^O, RH, ET and Soil_60_ using equal weights. No additional statistical fitting was used to optimise these weights, as the index was designed as a process-based composite metric rather than a predictive model. All calculations were performed using Addinsoft XLSTAT software version 2014.5.03 (Addinsoft Inc., New York, NY, USA).

## 3. Results

The combined dataset collected during the 2023 and 2024 growing seasons integrates isotopic, climatic and edaphic information at high temporal resolution (see Tables S1 and S2—Supplementary Material), enabling a mechanistic assessment of vine–atmosphere–soil interactions. Air temperature ranged between 7.9 °C in early spring and almost 29 °C in midsummer, while relative humidity (RH) fluctuated between 55% and 83%. Weekly evapotranspiration (ET) varied from 6.5 mm to over 43 mm, mirroring atmospheric demand. Soil matric potential at 60 cm depth spanned from values near field capacity (<10 cb) to severe dryness (>180 cb) during September 2024, indicating marked edaphic drought.

Isotopic compositions of precipitation, stem and leaf water showed consistent seasonal patterns. Leaf water was systematically more enriched in the heavy isotopes of hydrogen and oxygen compared with stem water, which in turn was slightly enriched relative to precipitation. Enrichment peaked during midsummer, coinciding with high temperature, low RH and high ET, and declined towards end of autumn as rainfall increased and soil water recharged. Leaf δ^13^C and δ^15^N followed similar seasonal tendencies, displaying higher (less negative) δ^13^C and higher δ^15^N values under dry, warm conditions, indicative of reduced stomatal conductance and limited nitrogen availability.

### 3.1. Dual-Isotope Patterns and Evaporative Enrichment

**Figure 3:**
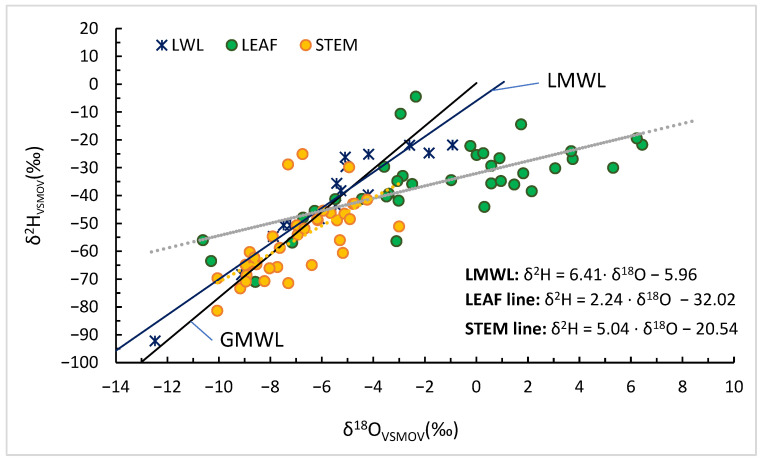
Dual-isotope (δ^2^H vs. δ^18^O) relationships for precipitation, stem and leaf water in *Vitis vinifera* cv. Sauvignon blanc at Orlești (2023–2024). The GMWL, LMWL and local evaporation (dashed line) lines (stem: orange; leaf: green) are shown.

The δ^2^H–δ^18^O relationships for *Sauvignon blanc* (Orlești, Vâlcea) during 2023–2024 (Figure 3) reveal a clear separation among precipitation, stem, and leaf water. Precipitation samples plot close to the Global Meteoric Water Line (GMWL; δ^2^H = 8 × δ^18^O + 10), reflecting the isotopic signature of local meteoric input with minimal fractionation. Stem water plots along a local evaporation line with a slope of approximately 5.0, lower than the GMWL, suggesting moderate evaporative enrichment and partial modification of xylem water. This intermediate behaviour likely reflects evaporation from near-surface soil layers before uptake and limited isotopic exchange within stem tissues.

Leaf water displays a much shallower regression slope of 2.24, typical of strong kinetic fractionation associated with transpiration. The pronounced flattening of this slope reflects rapid isotopic enrichment of leaf water through diffusion and equilibrium processes under conditions of low humidity and high evaporative demand. Slopes of 2–3 in leaf water are characteristic of conditions where kinetic fractionation dominates over equilibrium effects, typically under low relative humidity and enhanced evaporative demand [2,11,12]. Such shallow slopes emerge because hydrogen isotopes experience stronger kinetic limitation than oxygen isotopes during diffusion, resulting in a reduced δ^2^H/δ^18^O slope during partial evaporation [5]. The observed leaf water line (slope ≈ 2.24) is therefore consistent with Craig–Gordon predictions for Mediterranean summer conditions, where vapour pressure deficit and boundary-layer resistance exert strong control on isotopic enrichment [24].

Seasonal trajectories within the dual-isotope space further emphasise the interplay between source water and evaporative conditions. During early spring, both stem and leaf water closely follow the meteoric line, indicating uptake of freshly recharged soil moisture. As the season progresses and vapour pressure deficit increases, points migrate towards more enriched δ^18^O and δ^2^H values along the local evaporation lines. The progressive depletion of both isotopes observed in late summer and autumn corresponds to the transition to deeper, isotopically lighter soil water sources and cooler, more humid atmospheric conditions.

These isotope dynamics mirror the meteorological evolution of the growing season. Periods of strong δ^18^O enrichment in leaves coincide with high air temperature and low RH, confirming that leaf water isotopic composition is a sensitive integrator of atmospheric drought intensity. The consistent offset between stem and leaf isotopic signatures quantifies the degree of evaporative enrichment, linking canopy water status to environmental forcing.

The comparative slopes of the three water pools provide diagnostic information on the magnitude and mechanism of fractionation. The LMWL slope of 6.41 represents meteoric water; the stem line of ~5.0 reflects moderate evaporative enrichment, characteristic of xylem water partially influenced by isotopically fractionated soil moisture; and the leaf line of ~2.2 indicates intense transpiration-driven enrichment governed by kinetic effects. The observed hierarchy of slopes (6.41 > 5.04 > 2.24) illustrates the progressive enrichment from precipitation to stem to leaf, delineating the soil–plant–atmosphere continuum of isotopic modification.

### 3.2. Deuterium Excess (d) as an Indicator of Evaporative and Source-Water Dynamics

**Figure 4:**
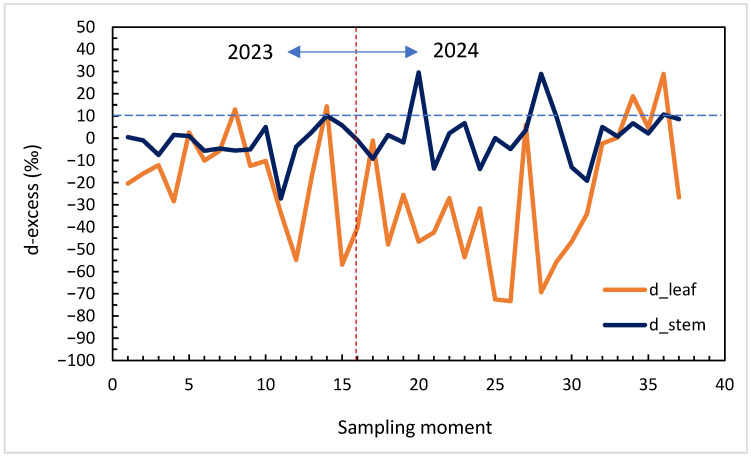
Time series of *d*-excess in leaf and stem water (2023–2024). The dashed horizontal line at +10‰ represents the Global Meteoric Water Line reference.

Across the 2023–2024 seasons, *d*-excess exhibited strong temporal variability and distinct signatures in stem and leaf water (Figure 4). Leaf *d*-excess ranged between approximately −70‰ and +10‰, with pronounced negative excursions during midsummer, coinciding with low relative humidity (RH < 60%) and high evapotranspiration (ET > 30 mm week^−1^). In contrast, stem *d*-excess values were more stable (−20‰ to +10‰), reflecting the isotopic buffering of xylem water derived from mixed and deeper soil sources.

Seasonal minima in leaf *d*-excess coincided with heat peaks and atmospheric dryness, confirming its high sensitivity to evaporative conditions. As humidity increased and soils rewetted in late summer and early autumn, *d*-excess values approached near-meteoric levels, indicating partial re-equilibration with atmospheric vapour and isotopic recharge of soil water.

The correlation analysis confirmed that leaf *d*-excess is strongly controlled by atmospheric variables (Table 1). A pronounced positive correlation with RH (r = +0.70, *p* < 0.001) and a significant negative correlation with ET (r = −0.55, *p* < 0.01) highlight the dominance of kinetic fractionation under dry conditions. Temperature also displayed a moderate negative relationship (r = −0.42, *p* < 0.05), reinforcing the link between high evaporative demand and isotopic disequilibrium.

In contrast, stem *d*-excess showed weak or insignificant correlations with these variables (|r| < 0.25), consistent with a slower turnover and integration of deeper soil water. This decoupling between leaf and stem isotopic signals underscores their complementary diagnostic roles: leaf *d*-excess reflects atmospheric drought, while stem *d*-excess traces source-water stability.

The isotopic behaviour of *d*-excess in grapevine water reveals two distinct components of drought stress. Strongly negative *d*-excess in leaves corresponds to intense kinetic fractionation during low RH and high ET, making it a sensitive isotopic indicator of atmospheric drought. In contrast, the stable *d*-excess observed in stems reflects the isotopic composition of the source-water reservoir, integrating soil moisture dynamics over time.

By quantifying the balance between equilibrium and kinetic processes, *d*-excess complements the δ^18^O and δ^2^H systems and strengthens the interpretation of evaporative enrichment. Incorporating *d*-excess into isotopic monitoring frameworks therefore enhances the capacity to diagnose rapid atmospheric drought events and long-term shifts in water-source dynamics within vineyard ecosystems.

Overall, the contrasting behaviour of *d*-excess in leaves and stems refines the interpretation of the dual δ^2^H–δ^18^O patterns. In leaves, the combination of high Δ^18^O and strongly negative d-excess marks periods when kinetic fractionation during transpiration is maximised by low RH and high ET, that is, when atmospheric drought is most intense, even before soil water is completely depleted. In stems, *d*-excess values remain closer to the meteoric reference and vary more slowly, indicating that xylem water behaves as a buffered mixture of soil water from different depths. Gradual changes in stem d-excess reflect progressive shifts in the source-water reservoir as the profile dries and roots rely more on deeper, less evaporatively modified layers. In this framework, Δ^18^O mainly quantifies the magnitude of leaf-level enrichment above xylem water, while d-excess helps to decide whether the observed isotope signal is dominated by rapid atmospheric forcing or by longer-term changes in soil water sources. This explains why leaf d-excess is tightly correlated with RH and ET, whereas stem d-excess shows only weak, non-significant relationships with short-term atmospheric drivers.

### 3.3. Relationships Between Isotopic Composition and Environmental Drivers

To quantify the environmental factors driving isotopic variability, the weekly δ^18^O and δ^2^H values of leaf and stem water were compared with air humidity (RH), evapotranspiration (ET), temperature, precipitation, and soil matric potential. The results reveal a consistent and physically coherent set of relationships (Table 2).

The Pearson correlation matrix reveals strong and systematic relationships between isotopic composition and environmental conditions, indicating clear climatic control on isotopic enrichment in grapevine water. The two isotopic systems (δ^2^H and δ^18^O) were tightly coupled both in leaves (r = 0.68) and stems (r = 0.68), reflecting common evaporative fractionation mechanisms. However, the weak relationship between leaf and stem δ^2^H (r = 0.21) points to additional enrichment at the leaf level, associated with transpiration-driven kinetic fractionation rather than direct isotopic transfer from xylem water.

Leaves exhibited a pronounced isotopic response to the atmosphere’s evaporative demand. The strong negative correlation between leaf δ^18^O and relative humidity (r = −0.69, *p* < 0.001) and its positive correlation with evapotranspiration (r = +0.56, *p* < 0.001) confirm that dry air and high ET promote rapid ^18^O enrichment in leaf water—a classical isotopic response to atmospheric stress. Air temperature exerted a secondary yet significant effect (r ≈ +0.43), primarily through its control of vapour pressure deficit and evaporative fluxes, while the influence of wind speed was negligible, indicating that boundary-layer effects were less important than ambient humidity in governing isotopic fractionation. Similar but weaker patterns were observed for δ^2^H_leaf, showing that hydrogen isotopes follow the same atmospheric trends with lower sensitivity.

**Figure 5:**
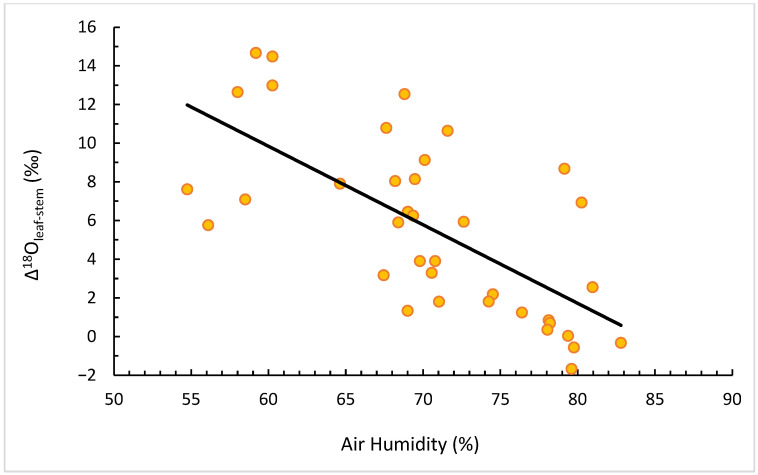
The correlation between Δ^18^O (leaf–stem) and RH.

When expressed as Δ^18^Oleaf–stem, the relationship with RH became even stronger (Figure 5), confirming that leaves add an evaporative enrichment on top of the xylem source-water signal. This enhanced contrast between leaf and stem isotopic values provides a quantitative measure of evaporative fractionation intensity and thus of short-term atmospheric drought.

In contrast, stem δ^2^H varied only weakly with weekly precipitation and soil matric potential at 60 cm (r ≈ −0.25), suggesting that xylem water integrates longer-term or mixed-depth soil water sources and responds more slowly to transient rainfall events. This buffered behaviour is consistent with the role of stem water as an indicator of source-water isotopic composition rather than of immediate evaporative demand.

Soil humidity at shallow depths (30 cm) correlated moderately with δ^18^O_leaf (r = +0.44), while deeper layers (60–100 cm) exhibited negative correlations with δ^2^H_leaf (r = −0.48), indicating a progressive shift in water uptake from shallow to deeper soil horizons under increasing dryness. The strong positive coupling between air and soil temperatures (r up to 0.93) and their inverse relationship with relative humidity (r ≈ −0.6) further underscore the synchronous evolution of thermal and evaporative stress.

Overall, the integrated isotopic–environmental analysis highlights a clear partitioning between rapid, atmosphere-driven isotopic responses in leaves and slower, soil-integrated isotopic signals in stems. Periods of low RH and high ET induced sharp ^18^O enrichment in leaf water, while stem δ^2^H remained comparatively stable, reflecting the isotopic buffering of deeper soil water.

Implications for drought: Both Δ^18^O (leaf–stem) and δ^18^O_leaf serve as sensitive indicators of atmospheric drought intensity (via RH or VPD), whereas δ^2^H_stem provides information on shifts in water sources or soil depths exploited by roots. When combined with vertical soil-moisture profiles, these relationships form the empirical foundation for constructing a vineyard Isotopic Drought Index (IDI) that integrates both atmospheric and edaphic components of water stress.

Lagged correlations (Table S3) between stem isotopes and soil matric potential at 60 cm (Soil_60_) did not reveal a stronger delayed response: for stem δ^2^H, r varied from −0.25 (lag 0) to −0.20 (lag 1) and −0.14 (lag 2), and for stem δ^18^O from −0.31 (lag 0–1) to −0.26 (lag 2). This supports the interpretation that stem water isotopes reflect a buffered source-water signal integrating soil moisture over time, rather than a sharply lagged response to individual weekly changes. In contrast, leaf isotopes showed slightly stronger associations with lagged Soil_60_ (e.g., δ^2^H_leaf, r = −0.48 at lag 0 vs. −0.57 at lag 2), consistent with the progressive development of edaphic drought in deeper layers. However, these correlations remained weaker than those with RH and ET, confirming that atmospheric demand is the primary control on short-term leaf-water enrichment.

**Figure 6:**
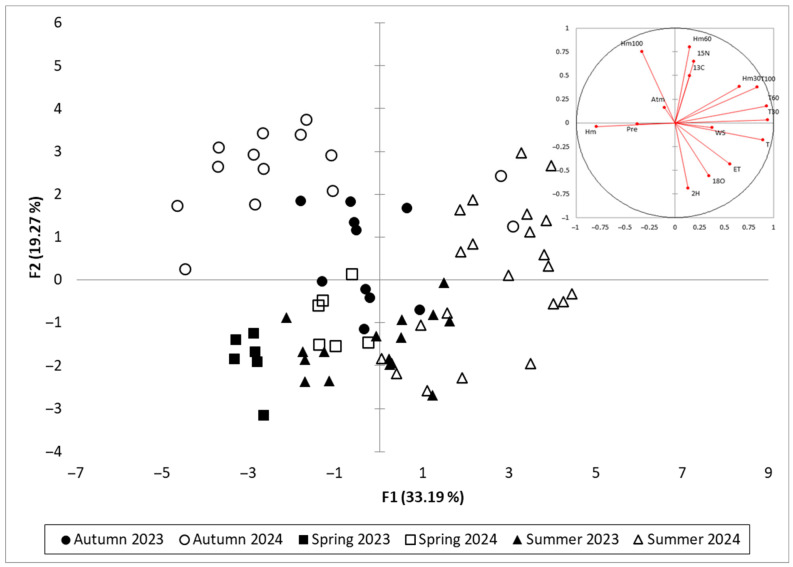
PCA projection plot of isotopic, climatic, and soil variables (inset) and seasonal sample observations (main plot) for *Vitis vinifera* cv. Sauvignon blanc in 2023–2024.

To assess the multivariate structure of the environmental and isotopic dataset, a principal component analysis (PCA) was performed using all weekly observations from the 2023 and 2024 growing seasons. The first two principal components (F1 and F2) together accounted for 52.46% of the total variance, with F1 explaining 33.19% and F2 accounting for 19.27% (Figure 6), as they summarise the main seasonal drought pattern in the dataset. Components with eigenvalues > 1 (F3–F5) raise the cumulative explained variance to 79.41%, but describe more specific, secondary structures. F3 (9.78%) is mainly related to wind speed, atmospheric pressure and ET, reflecting synoptic-scale variability rather than the primary drought gradient. F4 (9.11%) is dominated by additional variance in δ^2^H and δ^18^O, whereas F5 (8.06%) is largely controlled by precipitation and its impact on soil matric potential at 60 cm. These higher-order components add detail on atmospheric events and rainfall-driven recharge but do not modify the main contrast between thermal–evaporative drought (F1) and isotopic/edaphic responses (F2). Full eigenvalues for F1–F5 are reported in Table S4.

The first axis (F1) captured a dominant drought gradient, separating variables associated with increased evaporative demand and water stress from those indicative of wetter conditions. Strong positive loadings on F1 were observed for evapotranspiration (ET), air temperature (T), and soil matric potential at 30, 60, and 100 cm (Hm30, Hm60, Hm100), all of which increase under dry conditions. δ^13^C and δ^15^N were closely aligned with these variables, reflecting their association with physiological responses to drought stress, including reduced stomatal conductance and altered nitrogen cycling. In contrast, variables such as relative humidity (Hm), precipitation (Pre), and atmospheric pressure (Atm) loaded negatively on F1, representing cooler and more humid conditions conducive to lower isotopic enrichment.

The second axis (F2) appeared to distinguish atmospheric from soil-based influences, with positive contributions from δ^2^H, δ^18^O, wind speed (WS), and soil temperature across all depths (T30, T60, T100). This grouping suggests that F2 captures variation associated with evaporative enrichment, driven by kinetic fractionation under high temperature and wind exposure, particularly at the leaf level. The relative positioning of δ^2^H and δ^18^O further supports their role as tracers of short-term evaporative conditions, in contrast to δ^13^C and δ^15^N, which reflect longer-term physiological integration.

The spatial arrangement of variables in the biplot reinforces the functional distinctions among the isotope systems. δ^13^C and δ^15^N align with metrics of cumulative drought intensity, including ET and soil water tension, while δ^2^H and δ^18^O associate more strongly with atmospheric drivers and surface heat dynamics. Relative humidity and precipitation load in the opposite direction to the drought-associated variables, confirming their regulatory role in moderating evaporative enrichment. Among the soil variables, matric potential at 60 cm (Hm60) loads strongly along F1, supporting its use in the construction of the Isotopic Drought Index (IDI), as it captures effective root-zone water availability.

The corresponding PCA of seasonal observations shows clear separation by season along F1, with spring and autumn samples predominantly distributed in the negative F1 space, corresponding to higher humidity and lower evaporative demand. In contrast, samples from summer 2024 cluster in the positive F1 quadrant, reflecting elevated ET, higher δ^13^C and δ^15^N values, and increased soil water tension. The summer 2023 samples are more scattered, consistent with the milder and less sustained drought conditions observed that year. This temporal and seasonal separation confirms the ability of the PCA to resolve the progression of drought and associated physiological responses within the vineyard system.

### 3.4. Isotopic Drought Index (IDI)

**Figure 7:**
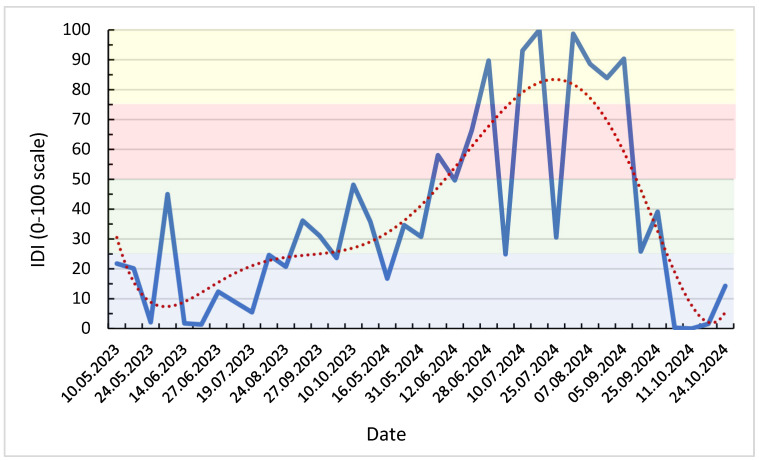
Weekly Isotopic Drought Index (IDI_0–100_) for 2023–2024, with LOWESS trend and drought severity thresholds (blue: <25 → humid conditions, green: 25–50 → moderate water stress, red: 50–75 → pronounced drought, yellow: >75 → severe drought).

To integrate the concurrent effects of atmospheric demand and soil water deficit, a composite Isotopic Drought Index (IDI) was computed from standardized variables: Δ^18^O_leaf–stem_, evapotranspiration (ET), air humidity (RH), and soil matric potential at 60 cm (Soil_60_) (Table 3). The index provides a normalized measure of drought intensity, combining isotopic, climatic, and edaphic information into a single dimensionless variable. Weekly IDI values for the 2023–2024 growing seasons (Figure 7) reveal clear temporal patterns reflecting the evolution of meteorological and soil conditions.

Low IDI values (<25) characterize the 2023 season, corresponding to wet and cool intervals with high RH (>75%) and low soil tension (<10 cb). These conditions resulted in limited isotopic enrichment (Δ^18^O < +6‰), consistent with efficient stomatal regulation and weak evaporative fractionation. Short fluctuations in June and October 2023 (IDI_0–100_ = 35–45) correspond to transient decreases in RH and moderate increases in ET.

By combining Δ^18^O_leaf–stem_, a direct indicator of Craig–Gordon evaporative enrichment, with environmental drivers such as RH, ET and soil water tension, the IDI metric effectively captures both atmospheric drought (via h) and soil water limitation. This aligns with conceptual frameworks linking leaf water enrichment to plant water stress in semi-arid ecosystems [12,25]. As a result, IDI provides a mechanistically grounded interpretation of the isotopic responses described below, enabling the distinction between short-term atmospheric drought and cumulative edaphic drought conditions. In contrast, 2024 exhibits a pronounced and sustained increase in IDI starting in late May, culminating in July–August with values exceeding 90 (equivalent to > +1.5 SD above the mean). This isotopic drought phase coincides with minimum RH (<60%), maximum evapotranspiration rates (>40 mm week^−1^), and soil water tensions surpassing 100 cb. The simultaneous enrichment of Δ^18^Oₗₑₐf_–_ₛₜₑₘ to > +14‰ indicates strong evaporative fractionation under conditions of limited soil water availability and reduced stomatal conductance.

Following the harvest period, IDI declined sharply during September–October 2024, as relative humidity increased and soil water potential partially recovered (Soil_60_ < 80 cb). The smoothed LOWESS trend (Figure 7) delineates a distinct seasonal trajectory: low isotopic drought intensity in 2023, followed by a single, high-magnitude drought pulse in the 2024 summer.

Overall, the IDI effectively integrates plant physiological response (Δ^18^O) with environmental drivers (RH, ET, Soil_60_), outperforming conventional meteorological drought metrics by directly quantifying plant-level water stress. Its temporal evolution provides a sensitive indicator of the onset, persistence, and recovery phases of vineyard drought.

### 3.5. Carbon and Nitrogen Isotope Dynamics

**Figure 8:**
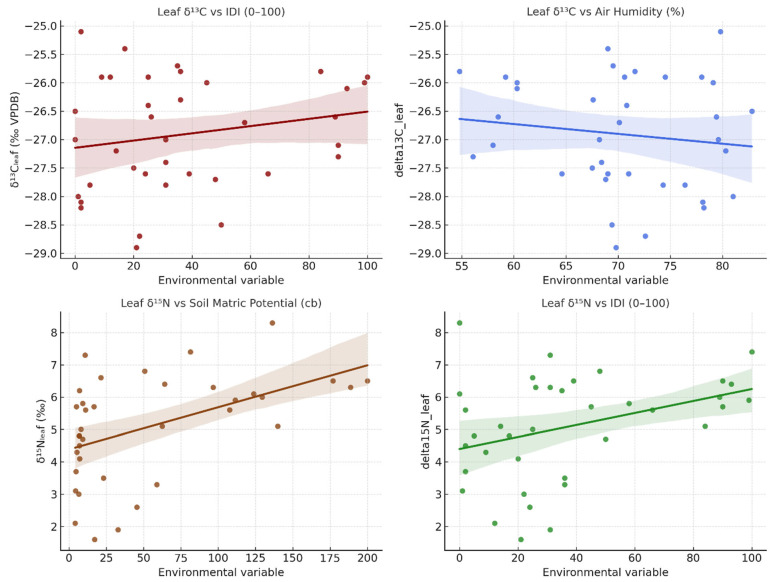
Relationships between δ^13^C, δ^15^N, and environmental drivers (RH, ET, Soil_60_, IDI) for leaves and stems of *Vitis vinifera* cv. Sauvignon blanc during 2023–2024.

The carbon (δ^13^C) and nitrogen (δ^15^N) isotopic compositions of vine leaves and stems provide complementary insights into the physiological adjustments of *Vitis vinifera* L. cv. Sauvignon blanc to water and nutrient stress during the 2023–2024 seasons (Figure 8). Leaf δ^13^C values ranged between −28.9 and −25.1‰ (VPDB), while stem δ^13^C ranged between −27.8 and −24.1‰, with stems consistently enriched by approximately 0.5–1.5‰ relative to leaves. Leaf δ^15^N values varied from 1.6 to 8.3‰, and stem δ^15^N from 2.0 to 9.5‰, the latter generally displaying higher enrichment in ^15^N, indicative of internal N remobilisation and fractionation during transport.

During the 2023 growing season, δ^13^C values were relatively depleted (more negative), consistent with open stomata, low intrinsic water-use efficiency (WUE), and limited evaporative stress, in agreement with low IDI values (<25) and high soil moisture (<10 cb). In 2024, δ^13^C gradually increased through May–August, reaching its least negative values (−25.1‰ in leaves, −24.1‰ in stems) at the peak of isotopic drought (IDI_0–100_ > 90). This enrichment reflects reduced stomatal conductance and enhanced WUE under intense atmospheric demand (RH < 60%) and severe soil desiccation (Soil_60_ > 100 cb).

Concomitantly, δ^15^N values rose steadily during the 2024 drought phase, particularly in stems, where they exceeded 8‰, reflecting restricted N uptake from dry soils and increased internal cycling. Such enrichment is typically associated with preferential uptake of ^15^N-enriched ammonium and greater isotopic fractionation linked to mineralisation and volatilisation under low soil moisture conditions. After rainfall in September–October 2024, both δ^13^C and δ^15^N declined, indicating a return to active gas exchange and N assimilation as soil water potential improved.

The offset between leaf and stem isotopic compositions highlights differences in integration timescales. Leaves respond rapidly to short-term changes in evaporative demand, whereas stems record longer-term physiological responses, reflecting the mixture of newly fixed and stored carbon and nitrogen pools.

Pearson correlation analysis confirms these relationships. Leaf δ^13^C was negatively correlated with relative humidity (r = −0.52, *p* < 0.01) and positively correlated with evapotranspiration (r = +0.43, *p* < 0.05), soil matric potential (r = +0.46, *p* < 0.05), and the isotopic drought index IDI (r = +0.49, *p* < 0.01). Stem δ^13^C exhibited similar, though slightly weaker, correlations (r = −0.44 with RH; +0.39 with Soil_60_; +0.41 with IDI). For nitrogen, leaf δ^15^N showed significant positive relationships with Soil_60_ (r = +0.48, *p* < 0.01) and IDI (r = +0.42, *p* < 0.05), while stem δ^15^N was even more strongly coupled (r = +0.56 and +0.53, respectively).

These results underline the integrated nature of δ^13^C and δ^15^N as mid-term physiological indicators. While δ^18^O and Δ^18^O respond to short-term evaporative conditions, δ^13^C and δ^15^N reflect cumulative responses to sustained drought, balancing carbon assimilation, stomatal control, and nitrogen metabolism. The concurrent enrichment of both isotopic systems during the 2024 summer drought demonstrates the coupling between carbon–water relations and nitrogen cycling under limited water availability.

These δ^15^N patterns should be interpreted as an integrated response of the soil–plant N cycle to drought, rather than as evidence for a single controlling process. Reduced soil water availability can alter organic matter mineralisation, nitrification and denitrification rates, restrict mass-flow and diffusive transport of NO_3_^−^ to the root surface, and modify gaseous and leaching losses of inorganic N. Together, these changes often lead to relatively ^15^N-enriched inorganic N pools and to greater reliance on internally recycled N, both of which can increase plant δ^15^N under dry conditions. In our case, the rise in leaf and stem δ^15^N with Soil_60_ and IDI is therefore best viewed as reflecting a drought-driven shift in N cycling and effective N acquisition, even though we did not directly measure soil NO_3_^−^/NH_4_^+^ pools or microbial processes.

Collectively, the isotopic evidence supports a two-phase model of vine drought response: (i) an immediate phase dominated by rapid evaporative fractionation captured by δ^18^O and IDI, and (ii) an integrative phase expressed in δ^13^C and δ^15^N, reflecting the longer-term adjustment of carbon and nitrogen metabolism. The coherence between δ^13^C, δ^15^N, and IDI highlights the diagnostic power of stable isotopes as a dual tracer system linking atmospheric stress, soil hydrology, and vine physiological regulation.

## 4. Discussion

### 4.1. Atmospheric Versus Edaphic Drought Signatures

The combined hydrogen and oxygen isotope evidence indicates that vine water isotopic composition was jointly driven by atmospheric evaporative demand and soil water availability. The hierarchy of δ^2^H–δ^18^O slopes—precipitation (≈6.4) > stem (≈5.0) > leaf (≈2.2)—clearly delineates progressive evaporative enrichment along the soil–plant–atmosphere continuum. Similar slope gradients have been reported in global syntheses [2], confirming that leaf water enrichment arises primarily from kinetic fractionation during transpiration, while stem water largely retains the isotopic fingerprint of its source.

The strong negative correlation between leaf δ^18^O and relative humidity (r = −0.69) and the positive relation with evapotranspiration (r = +0.56) confirm that atmospheric drought dominates short-term isotopic variability. These relationships are consistent with Craig–Gordon model predictions [8] and previous vineyard studies under Mediterranean climates [24,28]. In contrast, stem δ^2^H displayed delayed and dampened responses, reflecting the isotopic buffering from deeper, isotopically lighter water accessed by roots [29].

As the 2024 season advanced, soil drying (up to 200 cb at 60 cm) was accompanied by δ^2^H and δ^18^O depletion in stem water, signalling uptake from deeper horizons. This temporal decoupling between leaf and stem isotopic signals demonstrates how grapevines experience both transient atmospheric drought and cumulative edaphic drought, each leaving distinct isotopic imprints.

The application of the Craig–Gordon framework in this context follows standard practice in terrestrial plant isotope ecology. As in all field studies, several well-recognised assumptions—such as isotopic steady state, a well-mixed leaf water pool, and relatively stable boundary-layer conductance—may only be partially satisfied under natural diurnal variability [12,23]. These departures are typical and do not limit the interpretive value of the model. Instead, they imply that the observed dual-isotope relationships represent apparent, seasonally integrated expressions of Craig–Gordon processes. The consistency between our slope hierarchy, the strong δ^18^O–RH associations, and the negative leaf *d*-excess provides robust, mechanistically grounded evidence that the primary physical mechanisms described by the model are clearly expressed in this vineyard system.

### 4.2. Integrating Isotopic Indicators Through the IDI

The Isotopic Drought Index (IDI) effectively integrates key drivers of vine water status—relative humidity, evapotranspiration, soil matric potential, and isotopic enrichment (Δ^18^O)—into a single, normalised metric.

Weekly IDI values captured both the onset and peak of the 2024 midsummer drought, with the highest intensities between weeks 10–13 (July–August), where IDI_0_–_100_ exceeded 90, coinciding with low RH (<60%), ET > 35 mm week^−1^, and soil dryness above 100 cb.

This agreement between isotopic and environmental signals confirms the diagnostic sensitivity of IDI to both atmospheric and edaphic stress phases. The subsequent decline in IDI from September onward tracked soil rewetting after rainfall events, illustrating the capacity of IDI to represent both drought development and recovery.

Because the IDI explicitly incorporates Δ^18^O, it reflects actual plant water stress, not merely meteorological conditions. Such integration enhances interpretability compared with classical drought indices (e.g., SPI or SPEI), which lack a physiological component. Similar isotope-based drought indicators have been proposed for forest systems [25], but this study demonstrates their operational potential in viticulture, where precise irrigation timing and stress diagnostics are essential.

### 4.3. Carbon and Nitrogen Isotope Responses

The carbon and nitrogen isotope signatures complement water-isotope evidence by linking physiological adjustments and biogeochemical feedback.

Leaf δ^13^C values ranged from −28.9 to −25.1‰ and stem δ^13^C from −27.8 to −24.1‰, showing clear seasonal enrichment (less negative δ^13^C) during dry and high-IDI periods, indicative of reduced stomatal conductance and increased intrinsic water-use efficiency [14,29].

Leaf δ^13^C showed a clear negative relationship with relative humidity (r = −0.52, *p* < 0.01) and positive relationships with evapotranspiration (r = +0.43, *p* < 0.05), soil matric potential at 60 cm (r = +0.46, *p* < 0.05) and the isotopic drought index IDI (r = +0.49, *p* < 0.01). Stem δ^13^C displayed similar, though slightly weaker, correlations (r = −0.44 with RH; r = +0.39 with Soil_60_; r = +0.41 with IDI), supporting the view that δ^13^C is linked to drought intensity but integrates conditions over longer timescales than δ^18^O.

In contrast, leaf δ^15^N values (1.6–8.3‰) showed strong positive correlations with soil dryness (r = +0.48, *p* < 0.01) and IDI (r = +0.42, *p* < 0.05). Stem δ^15^N exhibited an even tighter link with soil moisture (r = +0.56) and IDI (r = +0.53), pointing to nitrogen enrichment under drought. This isotopic pattern is consistent with limited nitrogen uptake under restricted soil moisture, where mass flow and diffusion of nitrate decline, and preferential assimilation of ^15^N-enriched ammonium occurs. It is important to note that δ^15^N does not respond only to root uptake limitation, but to the combined effects of drought on N mineralisation and nitrification, gaseous and leaching losses, mycorrhizal transfer, and the relative use of NO_3_^−^ versus NH_4_^+^. Our measurements do not resolve these individual pathways, so the observed enrichment of δ^15^N under high Soil_60_ and IDI should be interpreted as a net signal of altered N cycling and reduced effective N acquisition under water stress, rather than a uniquely diagnostic mechanism.

Together, δ^13^C and δ^15^N form a complementary isotopic pair: δ^13^C integrates the plant’s photosynthetic response, whereas δ^15^N captures nutrient cycling and edaphic constraints.

### 4.4. Mechanistic Synthesis and Implications

The integrated isotope dataset outlines a mechanistic sequence of vine drought progression: early-season equilibrium with meteoric inputs → leaf δ^18^O enrichment under high ET and low RH → stem δ^2^H depletion during soil desiccation → and eventual δ^13^C and δ^15^N enrichment reflecting physiological and nutritional stress.

Taken together, the isotope data and environmental measurements point to a soil–plant–atmosphere system in which the different tracers respond over different effective timescales. Variations in δ^18^O and Δ^18^O closely track week-to-week changes in relative humidity and evapotranspiration, indicating that these signals mainly reflect short-term atmospheric drought. By contrast, δ^13^C and δ^15^N show stronger correspondence with the overall intensity of seasonal drought (IDI, Soil_60_) and with differences between the two study years, which is consistent with their use as integrative measures of cumulative carbon and nitrogen stress [30]. On this basis, we view δ^18^O as responding relatively quickly to atmospheric conditions, while δ^13^C and δ^15^N represent more integrated responses over longer periods. However, resolving the exact response times would require measurements at higher temporal resolution and specific time-series analyses [31,32,33].

The resulting Isotopic Drought Index provides a continuous measure linking these responses to environmental forcing. Its sensitivity to both fast (evaporative) and slow (soil-based) drought signals makes it suitable for real-time vineyard monitoring and precision irrigation. The distinction between “rapid” (δ^18^O) and “cumulative” (δ^13^C, δ^15^N) responses is based on relative coupling to weekly environmental drivers and on known differences in tissue turnover, rather than on explicit time-series modelling. Our weekly sampling resolution and two-year record do not allow us to identify a precise mechanistic transition point between these phases. Addressing this question would require higher-frequency isotope measurements and formal lag-response modelling, which we identify as an important avenue for future work.

### 4.5. Practical Implications for Precision Viticulture

The indicators developed in this work can be linked directly to vineyard management. In our case study, the Isotopic Drought Index (IDI) was rescaled to a 0–100 range and divided into four simple classes: IDI_0–100_ < 25 (humid conditions), 25–50 (moderate water stress), 50–75 (pronounced drought) and >75 (severe drought). In the Orlești vineyard, values above about 75 occurred during July–August 2024, when relative humidity was low, ET was high, soil tension at 60 cm exceeded 100 cb, and δ^13^C and δ^15^N were clearly enriched. Values below 25 characterised cool, humid periods with little isotopic enrichment and low soil tension.

For growers, these classes offer a straightforward way to read the combined isotope–meteorological information. Severe IDI values during sensitive stages such as flowering, fruit set or early berry growth would indicate that additional stress should be avoided (for example by delaying leaf removal, reducing crop load, or applying corrective irrigation where irrigation is available). By contrast, moderate IDI values (around 25–50) close to véraison may be acceptable, or even desirable, in vineyards aiming for high quality, as they reflect some increase in water-use efficiency without the extreme soil dryness observed at the peak of the 2024 drought.

Repeated IDI and isotope measurements over several seasons would also allow growers to identify blocks that systematically reach high IDI values and to distinguish whether they are mainly affected by atmospheric drought (strong Δ^18^O and very negative leaf d-excess) or by long-lasting soil water deficit (high Soil_60_ and δ^15^N enrichment). This information can support longer-term choices such as rootstock selection, soil organic-matter management or the design of an irrigation system. The absolute thresholds reported here are specific to the Orlești vineyard, but the same framework can be transferred to other sites and calibrated with local data.

In broader perspective, this multi-isotope framework (H–O–C–N) offers a process-based approach to drought diagnosis, bridging microclimate, soil, and plant physiology. Such integration is crucial under increasing climate variability, providing a mechanistic foundation for climate-resilient vineyard management and potentially extensible to forestry and agroecosystems.

## 5. Conclusions

This study demonstrates that the joint analysis of hydrogen, oxygen, carbon, and nitrogen isotopes provides a mechanistic and quantitative understanding of drought processes in vineyards. Dual δ^2^H–δ^18^O relationships confirm progressive enrichment from precipitation to stem and leaf water, driven primarily by atmospheric evaporative demand.

The IDI successfully integrates these controls—capturing timing, intensity, and recovery of drought events—and aligns closely with field observations of vine stress. Although the IDI thresholds presented here are specific to the Orlești site, the same isotope-based approach can be calibrated in other vineyards and combined with existing weather-station data to support irrigation planning and canopy management.

Meanwhile, δ^13^C and δ^15^N extend this interpretation, recording longer-term physiological and nutritional drought responses. Their combined dynamics reveal how isotopic systems trace both rapid atmospheric forcing and cumulative soil–plant feedback, offering a holistic framework for vineyard drought assessment.

This integrative approach thus provides a quantitative, transferable tool for precision viticulture and sustainable water management in a changing climate.

While the current study provides robust temporal resolution and analytical precision, several limitations should be acknowledged. First, spatial variability in soil properties and rooting depth may introduce heterogeneity not captured by single-site monitoring. Future work should integrate isotopic mapping and soil water extraction at multiple depths to resolve this variability. Second, the use of weekly sampling, though suitable for seasonal trends, may under-represent rapid isotopic fluctuations following rainfall or irrigation events. Continuous or automated water-vapour isotope monitoring would enhance temporal resolution. Finally, coupling isotopic indicators with physiological measurements such as sap flow, leaf water potential or chlorophyll fluorescence would strengthen causal inference between isotopic signals and vine function.

## Figures and Tables

**Table 1:** Pearson correlation coefficients (r) between *d*-excess and selected environmental variables (RH—Relative humidity; ET—Evapotranspiration; T—Temperature).

Relationship	r	*p*-Value	Interpretation
*d*_leaf—RH	+0.70	<0.001	Strong positive—enrichment decreases with increasing humidity
*d*_leaf—ET	−0.55	<0.01	Negative—high ET enhances kinetic fractionation
*d*_leaf—T	−0.42	<0.05	Moderate—warm, dry conditions lower *d*-excess
*d*_stem—RH	+0.18	0.31	Weak relationship—buffered isotopic signal
*d*_stem—ET	−0.20	0.28	Weak negative, non-significant
*d*_stem—T	−0.22	0.22	Weak, non-significant

**Table 2:** Pearson correlation coefficients between isotope variables (δ^18^O, δ^2^H, Δ^18^O) and environmental parameters (RH, ET, Temperature, Precipitation, Soil matric potential 30–100 cm).

	δ^2^H_leaf_	δ^18^O_leaf_	δ^2^H_stem_	δ^18^O_stem_	T	RH	W	P	Pp	ET	S_H_30	S_H_60	S_H_100	S_T_30	S_T_60	S_T_100
**δ^2^H_leaf_**	1.00	0.68	0.21	0.17	0.22	−0.35	0.11	−0.09	−0.24	0.36	0.10	−0.48	−0.47	0.10	0.04	−0.08
**δ^18^O_leaf_**	0.68	1.00	0.08	0.04	0.43	−0.69	0.42	−0.09	−0.24	0.56	0.44	−0.23	−0.39	0.40	0.24	0.23
**δ^2^H_stem_**	0.21	0.08	1.00	0.68	0.06	0.14	0.27	−0.07	0.10	0.32	−0.37	−0.25	−0.33	−0.03	−0.10	−0.20
**δ^18^O_stem_**	0.17	0.04	0.68	1.00	0.21	0.08	0.03	−0.39	0.08	0.21	−0.31	−0.31	−0.47	0.08	−0.01	−0.12
**T**	0.22	0.43	0.06	0.21	1.00	−0.61	0.19	−0.09	−0.24	0.68	0.35	−0.04	−0.53	0.93	0.85	0.68
**RH**	−0.35	−0.69	0.14	0.08	−0.61	1.00	−0.43	0.02	0.37	−0.41	−0.63	−0.19	0.23	−0.63	−0.61	−0.53
**W**	0.11	0.42	0.27	0.03	0.19	−0.43	1.00	0.07	0.04	0.30	0.21	0.06	−0.27	0.28	0.23	0.16
**P**	−0.09	−0.09	−0.07	−0.39	−0.09	0.02	0.07	1.00	0.03	0.06	−0.12	0.10	0.07	−0.13	−0.12	−0.17
**Pp**	−0.24	−0.24	0.10	0.08	−0.24	0.37	0.04	0.03	1.00	0.08	−0.28	0.04	−0.06	−0.16	−0.23	−0.26
**ET**	0.36	0.56	0.32	0.21	0.68	−0.41	0.30	0.06	0.08	1.00	0.00	−0.28	−0.56	0.58	0.45	0.22
**S_H_30**	0.10	0.11	−0.37	−0.31	0.35	−0.63	0.21	−0.12	−0.28	0.00	1.00	0.37	0.08	0.57	0.67	0.75
**S_H_60**	−0.48	−0.23	−0.25	−0.31	−0.04	−0.19	0.06	0.10	0.04	−0.28	0.37	1.00	0.70	0.16	0.29	0.46
**S_H_100**	−0.48	−0.39	−0.33	−0.47	−0.53	0.23	−0.27	0.07	−0.06	−0.56	0.08	0.70	1.00	−0.37	−0.21	0.04
**S_T_30**	0.10	0.40	−0.03	0.08	0.93	−0.63	0.28	−0.13	−0.16	0.58	0.57	0.16	−0.37	1.00	0.98	0.87
**S_T_60**	0.04	0.34	−0.10	−0.01	0.85	−0.61	0.23	−0.12	−0.23	0.45	0.67	0.29	−0.21	0.98	1.00	0.95
**S_T_100**	−0.08	0.23	−0.20	−0.12	0.68	−0.53	0.16	−0.17	−0.26	0.22	0.75	0.46	0.04	0.87	0.95	1.00

T—temperature, RH—relative humidity, W—wind speed, P—atmospheric pressure, Pp—precipitation, ET—evapotranspiration, S_H_30/60/100—soil humidity at 30/60/100 cm, S_T_30/60/100—soil temperature at 30/60/100 cm.

**Table 3:** Weekly Isotopic Drought Index (IDI) values and corresponding environmental variables (Δ^18^O, RH, ET, Soil60 cb) for *Vitis vinifera* cv. Sauvignon blanc (Orlești, Vâlcea, 2023–2024).

Date	Week	Δ^18^ O_leaf–stem_ (‰)	RH (%)	ET (mm)	Soil60 (cb)	IDI_raw_	IDI_0–100_
10 May 2023	1	5.93	72.63	20.77	6.64	−0.32	21.76
17 May 2023	2	3.17	67.45	18.49	7.13	−0.35	20.16
24 May 2023	3	0.84	78.13	23.08	6.98	−0.73	2.11
07 Jun 2023	4	8.68	79.15	44.08	4.98	0.16	45.02
14 Jun 2023	5	0.70	78.21	23.62	4.65	−0.74	1.76
21 Jun 2023	6	2.55	80.97	22.89	4.43	−0.75	1.40
27 Jun 2023	7	0.35	78.04	33.58	4.12	−0.52	12.38
05 Jul 2023	8	2.18	74.51	21.15	5.16	−0.59	8.88
19 Jul 2023	9	1.24	76.40	22.70	6.80	−0.66	5.47
02 Aug 2023	10	3.90	70.78	25.21	7.97	−0.26	24.65
24 Aug 2023	11	3.90	69.80	18.80	17.10	−0.34	20.74
14 Sep 2023	12	10.79	67.62	12.12	23.09	−0.02	36.17
27 Sep 2023	13	5.90	68.40	1.00	32.90	−0.13	31.07
04 Oct 2023	14	6.45	69.02	9.26	45.52	−0.28	23.64
10 Oct 2023	15	12.54	68.80	15.26	50.69	0.23	48.16
17 Oct 2023	16	10.64	71.59	11.31	58.79	−0.03	35.76
16 May 2024	1	1.33	69.00	22.00	6.80	−0.43	16.74
24 May 2024	2	8.14	69.48	22.40	6.98	−0.05	34.62
31 May 2024	3	8.04	68.19	16.80	10.80	−0.13	30.77
05 Jun 2024	4	9.13	70.11	41.05	9.20	0.43	58.06
12 Jun 2024	5	6.25	69.36	39.37	9.18	0.26	49.64
19 Jun 2024	6	7.90	64.63	43.20	11.13	0.61	66.39
28 Jun 2024	7	12.64	58.01	42.31	16.66	1.09	89.72
04 Jul 2024	8	3.29	70.56	24.20	21.29	−0.26	24.92
10 Jul 2024	9	14.48	60.26	35.68	64.10	1.16	93.09
18 Jul 2024	10	14.67	59.18	36.72	81.26	1.31	100.00
25 Jul 2024	11	1.81	74.25	24.30	96.60	−0.14	30.53
30 Jul 2024	12	12.98	60.26	35.68	111.40	1.28	98.72
07 Aug 2024	13	7.09	58.50	34.91	129.40	1.07	88.60
27 Aug 2024	14	7.61	54.75	22.50	139.86	0.97	83.90
05 Sep 2024	15	5.76	56.11	23.64	200.00	1.11	90.34
17 Sep 2024	16	0.03	79.38	15.14	188.70	−0.24	25.84
25 Sep 2024	17	1.80	71.03	13.07	176.90	0.04	39.13
07 Oct 2024	18	−1.68	79.61	6.46	136.25	−0.77	0.30
11 Oct 2024	19	−0.33	82.80	9.72	123.80	−0.77	0.00
16 Oct 2024	20	−0.57	79.76	10.29	107.55	−0.74	1.57
24 Oct 2024	21	6.93	80.26	12.63	62.50	−0.48	14.29

## Data Availability

All relevant data to the study are included in the article.

## Supplementary Materials

The following supporting information can be downloaded at: https://www.mdpi.com/article/10.3390/plants14243816/s1, Figure S1: Sensitivity analysis of the Isotopic Drought Index (IDI) to weighting structure. Five scenarios were tested: equal weights (W0) and ±25% increased weighting of Δ^18^O, RH, ET, and Soil_60_ (W1–W4). All scenarios reproduce the same seasonal pattern and peak drought intensity in summer 2024, demonstrating the robustness of the index; Table S1: Isotopic data of leaf and stem of *Vitis vinifera* cv. Sauvignon Blanc blanc from Orlești-Vâlcea (Romania), during 2023-2024 vintage; Table S2: Meteorological and soil measurements (Romania), during the sampling campaign (Orlești – Vâlcea, Romania; 2023-2024 vintage); Table S3: Pearson correlation coefficients between vine water isotopes and environmental variables, including 1–2 week lagged soil matric potential (Soil_30_, Soil_60_) for the 2023–2024 growing seasons; Table S4: Eigenvalues and percentage of explained variance for the first five principal components of the isotopic–environmental dataset (2023–2024).
