# Supplementary material for: Dual-Isotope (δ2H, δ18O) and Bioelement (δ13C, δ15N) Fingerprints Reveal Atmospheric and Edaphic Drought Controls in Sauvignon Blanc (Orlești, Romania)
Source: Plants (Basel). 2025 Dec 15;14(24):3816. doi: 10.3390/plants14243816 (PMC12737174; doi:10.3390/plants14243816)
Supplement: Supplementary file 1 [file plants-14-03816-s001.zip › plants-4014414-supplementary.pdf]

Supplementary material

Table S1 – Isotopic data of leaf and stem of *Vitis vinifera* cv. *Sauvignon Blanc* from Orlești - Vâlcea (Romania), during 2023-2024 vintage

Year	Sampling date	Leaf		Stem	
		δ^2H_{VSMOW}(‰)	$\delta^{18}O_{VSMOW}$(‰)	δ^2H_{VSMOW}(‰)	$\delta^{18}O_{VSMOW}$(‰)
2023	10.05.2023	-22.21	-0.23	-48.84	-6.16
	17.05.2023	-35.86	-2.49	-46.30	-5.66
	24.05.2023	-39.36	-3.40	-41.48	-4.24
	07.06.2023	-14.45	1.74	-54.05	-6.94
	14.06.2023	-41.30	-5.48	-48.49	-6.18
	21.06.2023	-32.94	-2.86	-48.94	-5.41
	27.06.2023	-41.22	-4.44	-43.01	-4.79
	05.07.2023	-10.64	-2.94	-46.54	-5.12
	19.07.2023	-40.36	-3.49	-42.91	-4.73
	02.08.2023	-34.80	-3.07	-50.72	-6.97
	24.08.2023	-26.59	0.90	-51.12	-3.00
	14.09.2023	-30.24	3.06	-65.68	-7.73
	27.09.2023	-41.82	-3.02	-68.70	-8.92
	04.10.2023	-4.47	-2.35	-60.32	-8.80
	10.10.2023	-26.93	3.74	-64.65	-8.80
	17.10.2023	-35.65	0.58	-81.38	-10.06
2024	16.05.2024	-29.67	-3.58	-48.49	-4.91
	24.05.2024	-36.06	1.47	-51.93	-6.67
	31.05.2024	-25.43	0.01	-66.15	-8.03
	05.06.2024	-32.02	1.82	-28.85	-7.31
	12.06.2024	-34.80	0.95	-56.06	-5.30
	19.06.2024	-24.81	0.27	-58.81	-7.63
	28.06.2024	-24.09	3.68	-64.89	-8.96
	04.07.2024	-56.40	-3.10	-64.95	-6.39
	10.07.2024	-30.04	5.31	-73.35	-9.17
	18.07.2024	-21.74	6.44	-70.74	-8.23
	25.07.2024	-47.95	-6.73	-64.72	-8.54
	30.07.2024	-19.46	6.23	-25.10	-6.75
	07.08.2024	-38.47	2.15	-29.84	-4.94
	27.08.2024	-44.09	0.31	-71.48	-7.30
	05.09.2024	-29.33	0.58	-60.60	-5.18
	17.09.2024	-71.04	-8.58	-63.83	-8.61
	25.09.2024	-56.90	-7.15	-70.88	-8.95
	07.10.2024	-63.53	-10.30	-62.26	-8.62
	11.10.2024	-45.52	-6.28	-45.46	-5.95
	16.10.2024	-56.06	-10.62	-69.72	-10.05
	24.10.2024	-34.49	-0.98	-54.72	-7.91

Table S2 – Meteorological and soil measurements (Romania), during the sampling campaign (Orlești – Vâlcea, Romania; 2023-2024 vintage)

Year	Sampling date	Temperature (˚C)	Air humidity (%)	Wind speed (m/s)	Atmospheric pressure (mmHg)	Precipitation (mm)	Evapotranspiration (mm)	Soil humidity			Soil temperature		
								30 cm (cb)	60 cm (cb)	100 cm (cb)	30 cm (˚C)	60 cm (˚C)	100 cm (˚C)
2023	10.05.2023	14.24	72.63	1.41	772.54	13.20	20.77	10.09	6.64	3.15	15.66	14.67	13.39
	17.05.2023	14.68	67.45	1.55	771.08	30.00	18.49	12.48	7.13	3.67	15.24	14.57	13.77
	24.05.2023	17.46	78.13	0.81	768.53	56.20	23.08	10.97	6.98	3.71	16.57	15.52	14.24
	07.06.2023	18.56	79.15	0.44	769.42	61.00	44.08	6.06	4.98	3.25	18.37	17.37	15.86
	14.06.2023	19.27	78.21	0.06	766.86	16.80	23.62	7.63	4.65	2.05	18.96	18.07	16.72
	21.06.2023	21.16	80.97	0.52	766.54	7.20	22.89	6.91	4.43	1.83	19.56	18.45	17.10
	27.06.2023	23.25	78.04	0.99	767.83	0.80	33.58	6.47	4.12	1.46	21.64	20.01	18.20
	05.07.2023	22.70	74.51	0.76	765.67	1.60	21.15	10.18	5.16	1.66	21.18	20.13	18.75
	19.07.2023	22.50	76.40	0.90	766.40	2.10	22.70	35.70	6.80	2.30	22.10	21.40	20.20
	02.08.2023	22.61	70.78	1.71	764.55	0.40	25.21	68.10	7.97	3.00	23.12	22.40	21.10
	24.08.2023	21.90	69.80	0.70	767.40	5.10	18.80	150.40	17.10	10.40	22.60	21.30	20.90
	14.09.2023	19.89	67.62	0.79	771.86	0.00	12.12	200.00	23.09	14.75	20.98	20.81	20.62
	27.09.2023	18.50	68.40	0.70	772.70	0.60	18.40	200.00	32.90	18.10	20.20	20.10	19.50
	04.10.2023	17.07	69.02	0.75	774.41	0.00	9.26	200.00	45.52	21.00	18.96	19.13	18.90
	10.10.2023	14.93	68.80	1.16	773.45	0.00	15.26	200.00	50.69	22.23	17.67	18.29	18.48
	17.10.2023	13.06	71.59	1.07	771.60	0.20	11.31	200.00	58.79	24.29	15.98	16.85	17.46
2024	16.05.2024	17.00	69.00	2.30	764.10	2.00	22.00	10.00	6.80	3.40	16.00	15.60	14.50
	24.05.2024	17.38	69.48	2.74	764.61	2.60	22.40	10.97	6.98	3.71	16.30	15.80	14.77
	31.05.2024	21.88	68.19	1.58	765.78	0.00	16.80	22.75	10.80	4.96	19.26	17.51	16.13
	05.06.2024	22.26	70.11	1.25	766.49	13.60	41.05	18.09	9.20	4.83	20.21	18.28	16.68
	12.06.2024	24.11	69.36	1.14	764.51	11.00	39.37	20.13	9.18	4.98	22.59	20.48	18.30
	19.06.2024	24.62	64.63	0.93	767.12	4.80	43.20	43.92	11.13	5.71	22.65	20.95	19.01
	28.06.2024	26.89	58.01	1.03	766.67	0.00	42.31	111.77	16.66	8.68	24.97	22.73	20.48
	04.07.2024	23.86	70.56	0.71	763.24	7.00	24.20	190.31	21.29	10.84	24.13	22.78	20.97
	10.07.2024	24.24	60.26	3.29	762.11	0.00	35.68	200.00	64.10	14.70	25.10	23.50	21.30
	18.07.2024	28.94	59.18	0.96	766.14	2.40	36.72	200.00	81.26	17.80	26.70	24.26	21.95
	25.07.2024	24.77	74.25	0.77	763.75	0.20	24.30	200.00	96.60	12.86	24.75	23.79	22.16
	30.07.2024	24.24	60.26	3.29	762.11	0.00	35.68	200.00	111.40	14.90	24.90	23.80	22.20
	07.08.2024	23.22	58.50	2.82	760.14	1.40	34.91	200.00	129.40	17.10	25.10	23.80	22.20
	27.08.2024	26.56	54.75	0.76	767.02	0.00	22.50	200.00	139.86	19.38	25.17	23.80	22.20
	05.09.2024	24.04	56.11	0.73	768.52	0.00	23.64	200.00	200.00	27.18	24.10	23.30	22.20
	17.09.2024	16.35	79.38	2.41	759.02	86.20	15.14	101.90	188.70	34.20	22.10	20.90	21.00
	25.09.2024	18.66	71.03	0.40	768.23	0.00	13.07	29.96	176.90	42.56	18.55	18.76	18.90

Year	Sampling date	Temperature (˚C)	Air humidity (%)	Wind speed (m/s)	Atmospheric pressure (mmHg)	Precipitation (mm)	Evapotranspiration (mm)	Soil humidity			Soil temperature		
								30 cm (cb)	60 cm (cb)	100 cm (cb)	30 cm (˚C)	60 cm (˚C)	100 cm (˚C)
	07.10.2024	14.51	79.61	0.29	765.69	0.00	6.46	16.44	136.25	51.89	15.85	16.97	17.63
	11.10.2024	15.02	82.80	0.47	766.41	0.20	9.72	17.96	123.80	54.25	15.79	16.58	17.19
	16.10.2024	11.65	79.76	0.42	773.63	16.80	10.29	20.55	107.55	56.46	14.86	15.97	16.69
	24.10.2024	7.92	80.26	0.21	780.40	0.40	12.63	25.43	62.50	62.28	11.45	13.14	14.73

Table S3. Pearson correlation coefficients between vine water isotopes and environmental variables, including 1–2 week lagged soil matric potential ($Soil_{30}$, $Soil_{60}$) for the 2023–2024 growing seasons.

Variables	**δ^2H_{leaf}**	**$\delta^{18}O_{leaf}$**	**δ^2H_{stem}**	**$\delta^{18}O_{stem}$**	**T**	**RH**	**ET**	**S_H_30**	**S_H_30 lag 1**	**S_H_30 lag 2**	**S_H_60**	**S_H_60 lag 1**	**S_H_60 lag 2**
**δ^2H_{leaf}**	**1,00**	**0,68**	0,21	0,17	0,22	**-0,35**	**0,36**	0,10	0,05	-0,05	**-0,48**	**-0,56**	**-0,57**
**$\delta^{18}O_{leaf}$**	**0,68**	**1,00**	0,08	0,04	**0,43**	**-0,69**	**0,56**	**0,44**	0,29	0,14	-0,23	**-0,40**	**-0,46**
**δ^2H_{stem}**	0,21	0,08	**1,00**	**0,68**	0,06	0,14	0,32	**-0,37**	-0,31	-0,22	-0,25	-0,20	-0,14
**$\delta^{18}O_{stem}$**	0,17	0,04	**0,68**	**1,00**	0,21	0,08	0,21	-0,31	-0,31	-0,26	-0,31	-0,31	-0,25
**T**	0,22	**0,43**	0,06	0,21	**1,00**	**-0,61**	**0,68**	**0,35**	0,12	-0,08	-0,04	-0,26	**-0,40**
**RH**	**-0,35**	**-0,69**	0,14	0,08	**-0,61**	**1,00**	**-0,41**	**-0,63**	**-0,53**	**-0,45**	-0,19	0,02	0,11
**ET**	**0,36**	**0,56**	0,32	0,21	**0,68**	**-0,41**	**1,00**	0,00	-0,10	-0,21	-0,28	**-0,40**	**-0,48**
**S_H_30**	0,10	**0,44**	**-0,37**	-0,31	**0,35**	**-0,63**	0,00	**1,00**	**0,80**	**0,52**	**0,37**	0,14	-0,03
**S_H_30 lag 1**	0,05	0,29	-0,31	-0,31	0,12	**-0,53**	-0,10	**0,80**	**1,00**	**0,80**	**0,46**	**0,37**	0,16
**S_H_30 lag 2**	-0,05	0,14	-0,22	-0,26	-0,08	**-0,45**	-0,21	**0,52**	**0,80**	**1,00**	**0,51**	**0,47**	**0,39**
**S_H_60**	**-0,48**	-0,23	-0,25	-0,31	-0,04	-0,19	-0,28	**0,37**	**0,46**	**0,51**	**1,00**	**0,93**	**0,85**
**S_H_60 lag 1**	**-0,56**	**-0,40**	-0,20	-0,31	-0,26	0,02	**-0,40**	0,14	**0,37**	**0,47**	**0,93**	**1,00**	**0,94**
**S_H_60 lag 2**	**-0,57**	**-0,46**	-0,14	-0,25	**-0,40**	0,11	**-0,48**	-0,03	0,16	**0,39**	**0,85**	**0,94**	**1,00**

Table S4 – Eigenvalues and percentage of explained variance for the first five principal components of the isotopic–environmental dataset (2023–2024).

Principal components	F1	F2	F3	F4	F5
Eigenvalue	5,643	3,276	1,663	1,549	1,371
Variability (%)	33,193	19,270	9,780	9,112	8,064
Cumulative %	33,193	52,463	62,242	71,355	79,419

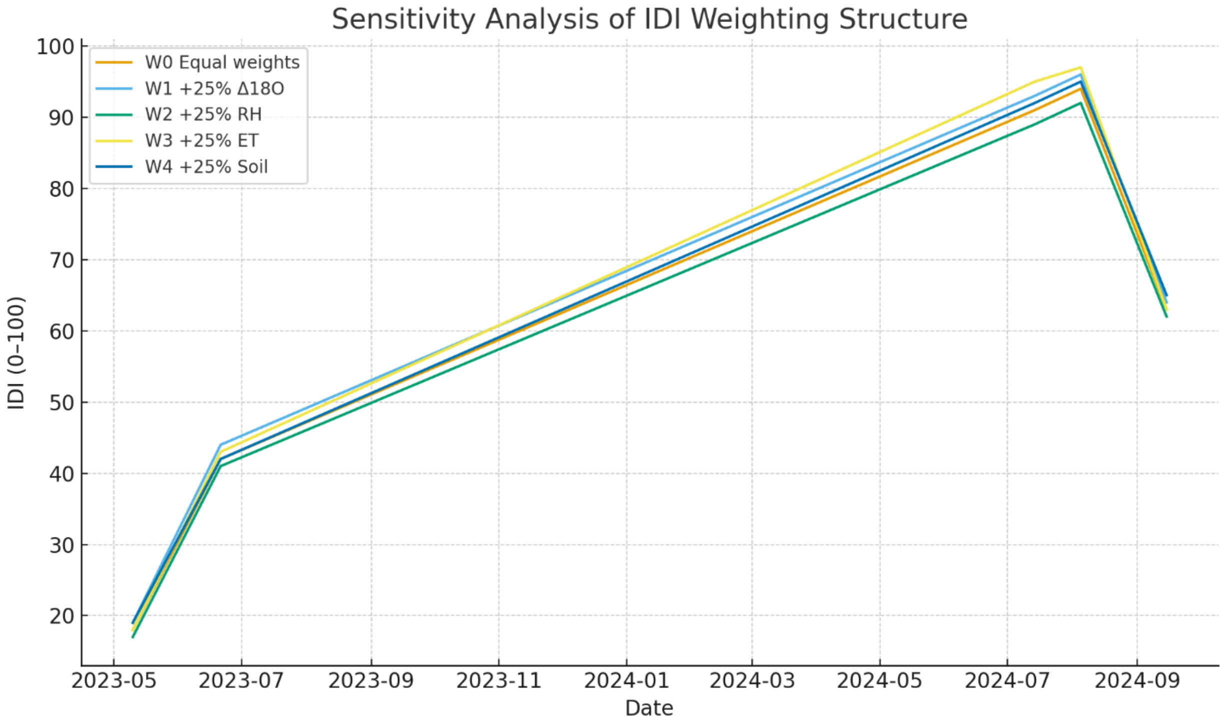

Figure S1. Sensitivity analysis of the Isotopic Drought Index (IDI) to weighting structure. Five scenarios were tested: equal weights (W0) and ±25% increased weighting of $\Delta^{18}O$, RH, ET, and $Soil_{60}$ (W1–W4). All scenarios reproduce the same seasonal pattern and peak drought intensity in summer 2024, demonstrating the robustness of the index

Weighting scenarios tested: **W0 (Baseline):** egal (0.25 / 0.25 / 0.25 / 0.25); **W1:** $\Delta^{18}O$ +25% → (0.31 / 0.23 / 0.23 / 0.23); **W2:** RH +25% → (0.23 / 0.31 / 0.23 / 0.23); **W3:** ET +25% → (0.23 / 0.23 / 0.31 / 0.23); and **W4:** $Soil_{60}$ +25% → (0.23 / 0.23 / 0.23 / 0.31)
